# Influence of 4,4’-azobis (4-cyanopentanoic acid) in Transmission and Reflection Gratings Stored in a PVA/AA Photopolymer

**DOI:** 10.3390/ma9030194

**Published:** 2016-03-15

**Authors:** Elena Fernandez, Rosa Fuentes, Augusto Belendez, Inmaculada Pascual

**Affiliations:** 1 Dep. Optica, Farmacología y Anatomía, Universidad de Alicante, Apartado de correos 99, Alicante E-03080, Spain; fuentes@ua.es (R.F.); pascual@ua.es (I.P.); 2 Dep. Física, Ingeniería de Sistemas y Tª de la Señal, Universidad de Alicante, Apartado de correos 99, Alicante E-03080, Spain; a.belendez@ua.es

**Keywords:** holography, photopolymers, holographic recording materials

## Abstract

Holographic transmission gratings with a spatial frequency of 2658 lines/mm and reflection gratings with a spatial frequency of 4553 lines/mm were stored in a polyvinyl alcohol (PVA)/acrylamide (AA) based photopolymer. This material can reach diffraction efficiencies close to 100% for spatial frequencies about 1000 lines/mm. However, for higher spatial frequencies, the diffraction efficiency decreases considerably as the spatial frequency increases. To enhance the material response at high spatial frequencies, a chain transfer agent, the 4,4’-azobis (4-cyanopentanoic acid), ACPA, is added to the composition of the material. Different concentrations of ACPA are incorporated into the main composition of the photopolymer to find the concentration value that provides the highest diffraction efficiency. Moreover, the refractive index modulation and the optical thickness of the transmission and reflection gratings were obtained, evaluated and compared to procure more information about the influence of the ACPA on them.

## 1. Introduction

Photopolymers are materials whose main components are organic polymers, and they are mainly characterized for being sensitive to light with a particular wavelength. The basic formulation includes a sensitizing dye, an initiator which is a free radical generator, and one or more polymerizable monomers. These components are placed in a binder such as polyvinyl alcohol, polyacrylate, polyvinylchloride, *etc.* These types of materials absorb light of a certain wavelength that excites the dye and activates the initiator. This substance generates free radicals, which react with the monomer to produce a polymerization reaction. The polymer chains generated in the photopolymerization reaction are responsible for the properties of these materials [1].

These materials have many applications, such as the manufacture of photonic lattice structures [2], photo-curable resins [3] or polymer waveguides [4] among others. This work will focus on their application as holographic recording materials.

Holography is an optical technique that allows the recording of an object wavefront (in amplitude and phase) in the material and its reconstruction. The recording is performed through the interference of the light beam coming from the object and a reference light beam. The interference of these two beams produces a pattern formed by “bright” and “dark” fringes, which modify the holographic recording material generating the hologram. Thus, the hologram is stored by means of the variation of certain material properties. When the hologram is illuminated with the reference beam, an identical wavefront to the object that was recorded is obtained.

Obtaining holograms of three-dimensional objects has sparked great worldwide interest. Among the different applications of holography, we can highlight holographic interferometry, which can be used to obtain information from some material properties [5], the manufacture of lenses [6,7], holographic optical elements (HOEs) [8,9,10] or data encryption [11].

However, one of the fields of holography that has had more importance in recent years is the manufacturing of holographic memories [12,13,14]. Our society has gotten used to generating a huge amount of information that must be processed and stored. One possibility to increase the storage density is to use not only the surface but also the entire material volume, which is directly achieved with holographic storage. If the stored objects are data pages, the holographic storage of all of them constitutes a holographic memory.

To increase the amount of holographically stored data, it is necessary that the object bits are as small as possible, so the material must be capable of resolving high spatial frequencies. However, the diffraction efficiency in photopolymers decreases as the spatial frequency increases, since they cannot resolve high spatial frequencies correctly [15]. For this reason, in this work, a chain transfer agent named 4,4’-azobis (4-cyanopentanoic acid) (ACPA) is added to a polyvinyl alcohol (PVA)/acrylamide (AA) based photopolymer in order to increase the diffraction efficiency of the recorded gratings when high spatial frequencies are recorded in the material. In previous works, the proper functioning of materials containing ACPA in their composition has been demonstrated. Ortuño *et al.* [16] achieved a low noise level in 900 μm PVA/AA photopolymers, obtaining gratings with a maximum diffraction efficiency above 85% by using ACPA. This had a great significance considering that it was the first time that diffraction efficiencies as high as 80% had been obtained for 900 μm PVA/AA photopolymer layers. This result is due to the effect induced by ACPA thanks to the small size of the polymer chains. The photopolymer with ACPA has a high fraction of low molecular weight polymer chains, which allow a better definition of the three-dimensional sinusoidal contour of the fringes in the diffraction grating, thus leading to higher diffraction efficiencies. Moreover, the incorporation of ACPA has been used to improve the stability of high spatial frequency diffraction gratings [15]. In this sense, the ACPA improved the fixation of the gratings, since the composition with ACPA experienced a smaller decrease of the diffraction efficiency after a curing process.

The standard photopolymer is composed by acrylamide (AA) as the polymerizable monomer, triethanolamine (TEA) as radical generator, polyvinyl alcohol (PVA) as binder and yellowish eosin (YE) as sensitizer. In addition, different concentrations of ACPA are incorporated into this composition to analyze the response of the material when gratings with high spatial frequencies (2658 lines/mm and 4553 lines/mm) are recorded in it. Once the diffraction efficiency is obtained, the experimental values are fitted using the Couple Wave Theory [17] to obtain grating parameters such as the refractive index modulation and the optical thickness, which provide an indicator of the grating improvement with the incorporation of the ACPA into the photopolymer.

## 2. Chemical Process

The chemical processes that take place in the photopolymer materials are essentially initiation, propagation and termination.

During the initiation process, the photopolymer is illuminated by a wavelength at which the material is sensitive. Then, the initiator system formed by the dye and the radical generator absorbs photons and produces free radicals, which are combined with the monomers present in the photopolymer to generate chain initiators [18]:

(1) $$I\overset{hv}{\to }2R^{•}$$

(2) $$R^{•}+M\overset{k_{i}}{\to }M^{•},$$

where *I* is the initiator system, *hν* indicates the energy absorption from the photons, *R*^•^ is the initiator of free radicals, *M* is the monomer, *k_i_* is the chain initiation kinetic constant and *M*^•^ is the chain initiator.

During the propagation process, the chain initiators are bound to other monomer molecules to form longer chains, *i.e.*, a chain initiator *M^•^_1_* is bound to a molecule *M* to form another longer chain *M^•^_2_* and so on.

(3) $$\begin{array}{c} M_{1}^{•}+M\overset{k_{p}}{\to }M_{2}^{•}\\ \vdots \\ M_{n}^{•}+M\overset{k_{p}}{\to }M_{n+1}^{•} \end{array}$$

where *k_p_* is the rate constant of propagation. Through the propagation process, a polymer chain is generated.

During the termination process, the macro-radical chains of polymer are combined together to finish the reaction according to the following equations:

(4) $$M_{x}^{•}+M_{y}^{•}\overset{k_{tc}}{\to }M_{x+y}^{•},$$

(5) $$M_{x}^{•}+M_{y}^{•}\overset{k_{td}}{\to }M_{x}+M_{y},$$

where *k_tc_* and *k_td_* are the rate constants of combination and disproportionation termination respectively. 

Moreover, in this work an additional chemical process occurs as a result of the incorporation of a chain transfer agent (CTA) in the photopolymer composition: the chain transfer mechanism. The CTA produces a premature termination in the growth of macro-radicals in the propagation process (*M*^•^_*n*_) and generates a new radical *A*^•^ that can start a new polymer chain by reacting with a monomer molecule. The chain transfer reactions can be written as:

(6) $$M_{n}^{•}+XA\overset{k_{tr}}{\to }M_{n}-X+A^{•}$$

(7) $$A^{•}+M\overset{k_{a}}{\to }M_{1}^{•}$$

where *XA* is the CTA, *A*^•^ is the new radical produced in the reaction and *k_tr_, k_a_* are the kinetic constants of the processes.

Due to premature termination of the reaction (as a result of the presence in the material of the CTA), the polymer chains stop growing and therefore chains become shorter. Moreover, thanks to the generation of new radicals, other chains can be formed and thus the polymerization rate does not decrease.

Therefore, the main effect to be achieved by introducing the CTA in the photopolymer composition is to reduce the length of the polymer chains in order to improve the material response when high spatial frequency gratings are recorded.

## 3. Materials and Methods 

### 3.1. Preparation of the Material

The holographic gratings were recorded in a PVA/AA based photopolymer. The main components of this material are AA as a polymerizable monomer, PVA as binder, TEA as radical generator, and YE as sensitizer. The TEA and the YE establish the initiator system. Table 1 shows the concentrations of the components for this photopolymer (C1). 

Composition C1 provides diffraction efficiencies around 90% for holographic transmission gratings recorded with a spatial frequency of 950 lines/mm [15]. However, when the spatial frequency is increased in the same composition, the diffraction efficiency of the gratings is not above 5% for gratings with a spatial frequency of 4600 lines/mm [15]. Therefore, it was decided to add the ACPA as a CTA to composition C1, for which the main effect is a reduction in the length of the polymer chain (as explained in Section 2) in order to improve the material response at higher spatial frequencies. In order to optimize the composition and obtain gratings with maximum diffraction efficiency, four different concentrations of ACPA between 0.006 M and 0.015 M (C2 to C5 in Table 1) were added to the photopolymer C1. 

**Figure 1:**
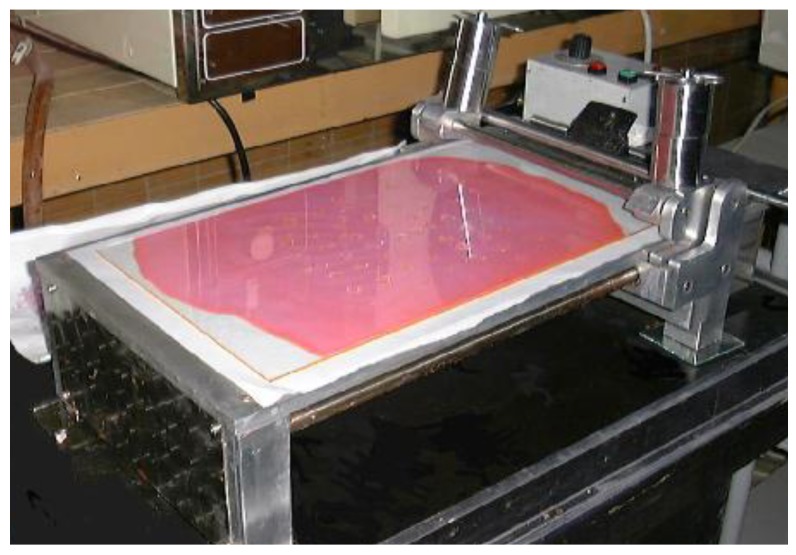
Photograph of the photopolymer material after its manufacturing.

To obtain the photopolymers with the compositions given in Table 1, a solution of PVA in water was first prepared by heating, and once all the PVA had been dissolved, it was allowed to cool. Simultaneously, a solution of AA and TEA was prepared in water. These two components were mixed in the necessary proportions to obtain the concentrations of composition 1 (Table 1). Finally, YE was added to the solution under red light, since the material is not sensitive at this wavelength. In the case of the compositions C2 to C5 indicated in Table 1, the ACPA was also added to the solution and stirred until it was completely dissolved. The mixture was then deposited by gravity on a glass plate, and it was left in darkness for approximately 24 h to allow the water to evaporate. This process was carried out at a temperature *T* = 20 °C and relative humidity *RH* = 40% in order to obtain samples with a physical thickness of 100 ± 5 μm. Layers with such physical thickness will be used to record transmission and reflection gratings in the following sections. Figure 1 shows a photograph of the sample that was used to store the gratings. Once dry, the glass was cut into squares of 6 cm × 6 cm. 

After manufacturing the material, the refractive index and the absorption spectrum were measured.

The refractive index of the material was measured through an Abbe’s refractometer, and a value *n* = 1.506 ± 0.003 was obtained for the compositions C1 to C5 indicated in Table 1. This refractive index will be used to calculate the spatial period of the recorded gratings and the Bragg wavelength in Section 3.2. 

**Figure 2:**
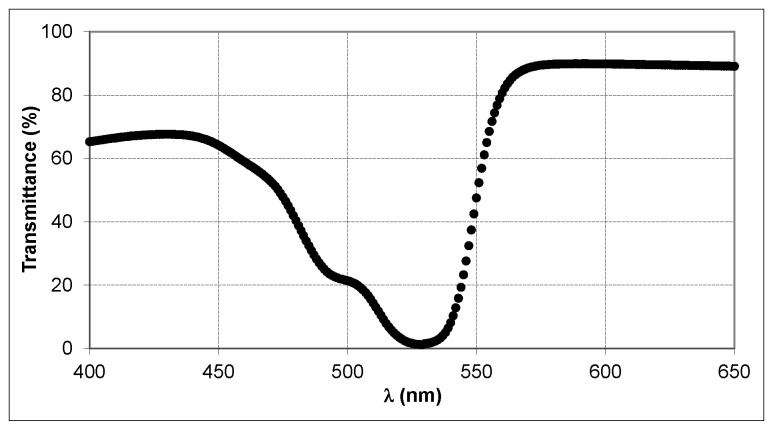
Transmission spectrum of the unexposed photopolymer plate.

Figure 2 shows the transmission spectrum *versus* the wavelength for the materials given in Table 1. The plot exhibits a minimum transmission (*i.e.*, maximum absorption) at the wavelength *λ* = 532 nm, which is the recording wavelength of the laser. In the range between *λ* = 570 nm and *λ* = 650 nm, the transmittance is at a maximum and thus the material does not absorb this range of wavelengths or absorbs very little. The geometries for the grating recording and reconstruction were chosen so that the Bragg angle (in transmission geometry) or Bragg wavelength (in reflection geometry) would appear in this non-absorbing spectral range (see Section 3.2.). 

### 3.2. Holographic Setup

In this work, gratings with a spatial frequency of 2658 lines/mm were recorded with a holographic transmission setup, whereas gratings with a spatial frequency of 4553 lines/mm were recorded with a holographic reflection setup. 

**Figure 3:**
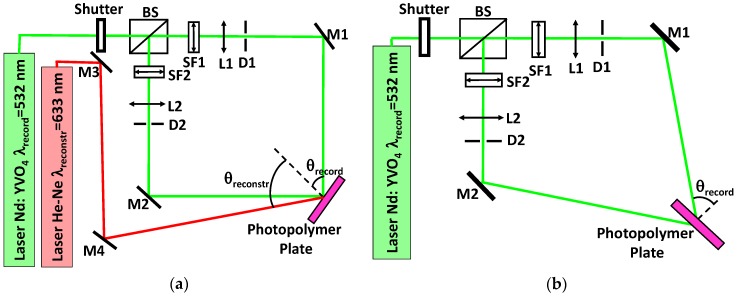
(**a**) Experimental setup for transmission gratings; (**b**) experimental setup for reflection gratings. Mi: mirrors, BS: beam splitter, Li: lenses, SFi: microscope objective lens and pinhole, Di: diaphragms, *θ_record_* is the angle with which the recording laser beams impinge in the material, *θ_reconstr_* is the angle with which the reconstruction laser beam impinges in the material.

During the recording stage, in both setups, the object beam and the reference beam interfered within the material impinging on it at an angle θ with respect to the normal of the film plane. The laser used in the recording stage was an Nd:YVO4 laser (Coherent Verdi V2) emitting a beam with a wavelength of 532 nm, at which the material is sensitive (Figure 2). All the laser beams have linear polarization perpendicular to the plane of incidence, which allows an optimal interference since the interfering electric fields of the recording beams are parallel. The laser beam was split into two beams with a beam-splitter, and the beams were expanded, collimated and directed to the material with the elements showed in Figure 3a (transmission setup) and Figure 3b (reflection setup).

The reconstruction stage is different for transmission and reflection holograms due to the geometry of both holographic systems. 

In the reconstruction of holographic transmission gratings, an He-Ne laser with a wavelength of 633 nm was used because the material is not sensitive at such a wavelength, as it can be seen in Figure 2. The diffraction efficiency of the transmission gratings was defined as the ratio of the power of the diffracted beam to the power of the incident beam. Furthermore, the diffracted beam and the reconstruction beam have linear polarization perpendicular to the plane of incidence.

In the case of holographic reflection gratings, the diffraction efficiency in the reconstruction stage was measured with a double beam spectrophotometer, which measures the photopolymer transmittance *versus* the wavelength. Consequently, unpolarized white light at normal incidence upon the sample was used in this case. The He-Ne laser could not be used to reconstruct the reflection grating since the diffracted beam overlaps the reflected beam at the air-material interface because of the symmetric geometry.

When the reflection grating is stored in the photopolymer, an additional peak located at the Bragg wavelength appears in the transmittance curve, and this may be used to calculate its diffraction efficiency. Once the transmittance is known, the diffraction efficiency (*DE*) is defined as the depth of the transmittance peak and can be calculated from Equation (8) [19]:

(8) $$DE=\frac{T_{p}-T_{pg}}{T_{p}}$$

where *T_p_* is the transmittance of the photopolymer layer without the recorded grating and *T_pg_* is the transmittance of the photopolymer layer with the recorded grating.

In the holographic transmission setup, a recording angle of *θ_record_* = 45° was chosen to achieve a spatial frequency as high as possible in this geometry. According to Bragg’s Law for the transmission geometry (Equation (9)), a theoretical spatial period of *Λ_th_* = 0.3762 μm which corresponds to a spatial frequency (1/*Λ_th_*) of 2658 lines/mm was obtained with a recording wavelength of *λ_record_* = 532 nm. In the reconstruction stage, the wavelength used to reconstruct the grating was *λ_reconst_* = 633 nm, which, according to Equation (9), allowed for obtaining a reconstruction angle (in the air) *θ_reconstr_* = 57.3°. This reconstruction angle is the Bragg angle, at which the maximum diffraction efficiency (*DE_Max_*) is obtained:

(9) $$\Lambda_{th}=\frac{\lambda }{2\sin\theta }$$

In the holographic reflection setup, a recording angle of *θ_record_* = 63.3° was chosen to record the gratings in this geometry. According to Bragg’s Law for the reflection geometry (Equation (10)), a theoretical spatial period of *Λ_th_* = 0.2194 μm which corresponds to a spatial frequency (1/*Λ_th_*) of 4558 lines/mm was obtained with a recording wavelength of *λ_record_* = 532 nm using a photopolymer refractive index of *n* = 1.506 (Section 3.1). In the reconstruction stage, the stored hologram was placed perpendicular to the spectrophotometer beam (*θ_reconstr_* = 0°). Taking into account the previous values for the refractive index, spatial period and reconstruction angle, we found that the diffraction peak theoretically appears around a Bragg wavelength of 660 nm, at which the maximum diffraction efficiency (*DE_Max_*) is obtained. Consequently, such a diffraction peak does not overlap with the absorption peak, as it is inferred from Figure 2:

(10) $$\Lambda_{th}=\frac{\lambda }{2\;\sqrt{n^{2}-\sin^{2}\theta }}$$

### 3.3. Fitting Procedure through Coupled Wave Theory

According to Kogelnik’s Coupled Wave Theory [17], the *DE* of a non-slanted reflection or transmission grating is given by:

(11) $$DE=\frac{\sin^{2}\left(\sqrt{\upsilon^{2}+\xi^{2}}\right)}{1+{\left(\frac{\xi }{\upsilon }\right)}^{2}}$$

where *υ* and *ξ* are obtained as:

(12) $$\upsilon =\frac{\pi \cdot \Delta n\cdot d}{\lambda_{reconstr}\cdot \cos\left({\theta \prime }_{reconstr}\right)}$$

(13) $$\xi =\frac{\pi \cdot d}{\Lambda \cdot \cos\left({\theta \prime }_{reconstr}\right)}\left[\left|\sin\left({\theta \prime }_{reconstr}\right)\right|-\frac{\lambda_{reconstr}}{2\cdot n\cdot \Lambda }\right]$$

In Equations (12) and (13), *Δn* is the refractive index modulation, *d* is the optical thickness, *λ_reconstr_* is the reconstruction wavelength, *θ*^′^*_reconstr_* is the reconstruction angle inside the sample (obtained from *θ_reconstr_* through Snell law), *λ_reconstr_* is the reconstruction wavelength in the air, *n* is the average refractive index of the sample and *Λ* is the grating period.

For transmission gratings, the *DE* is measured as a function of *θ_reconstr_*. Thus, knowing that the average refractive index is *n* = 1.506 and that the reconstruction wavelength is *λ_reconstr_* = 633 nm, the parameters *Δn*, *d* and *Λ* are fitted through Equations (11)–(13). For reflection gratings, DE is measured as a function of the reconstruction wavelength *λ_reconstr_*. In this case, the known parameters are the refractive index *n* = 1.506 and the reconstruction angle *θ_reconstr_* = 0°, from which *Δn*, *d* and *Λ* are fitted through Equations (11)–(13).

### 3.4. Shrinkage Measurement Procedure

In hydrophilic materials like the one presented in this work, the evaporation of the water contained in its composition can produce a volume reduction that leads to a change in the spatial period of the grating. This phenomenon is known as shrinkage. 

Evidence that a material has shrinkage occurs when the experimental period of the grating recorded in the sample, *Λ_exp_*, is smaller than that defined by the geometrical conditions of the recording, *Λ_th_*. In other words, the material shrinkage induces a change in the fringe spacing *ΔΛ* = *Λ_th_* − *Λ_exp_*. The experimental period of the grating *Λ_exp_* can be obtained from the fitting of the experimental data with the Coupled Wave Theory (detailed in the previous section).

Criante *et al.* [20] define the shrinkage as the ratio between the shift of the spatial period that is observed and the theoretical spatial period of the grating if there was not shrinkage, *i.e.*,:

(14) $$S=\frac{\Lambda_{th}-\Lambda_{\exp}}{\Lambda_{th}},$$

In Section 4, the shrinkage is calculated through Equation (14) for all the transmission and reflection gratings presented in this work. 

## 4. Results and Discussion

In this section, the results obtained from recording holographic transmission and reflection gratings are presented.

**Figure 4:**
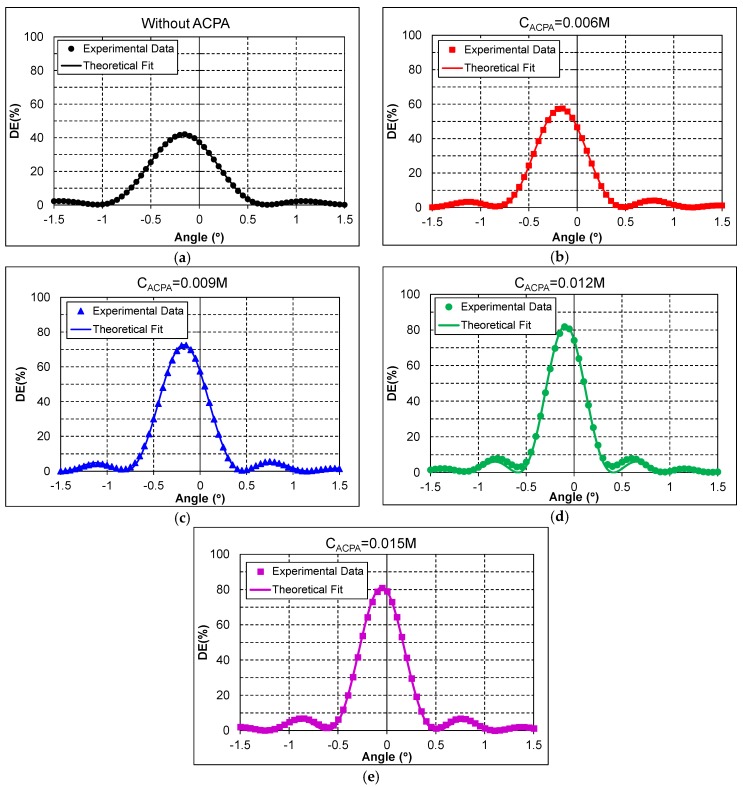
*DE* as a function of the reconstruction angle for transmission gratings stored with a spatial frequency of 2658 lines/mm for the compositions given in Table 1, (**a**) C1 without ACPA; (**b**) C2 with *C_ACPA_* = 0.006 M; (**c**) C3 with *C_ACPA_* = 0.009 M; (**d**) C4 with *C_ACP_* = 0.012 M; and (**e**) C5 with *C_ACPA_* = 0.015 M.

Firstly, transmission gratings with a spatial frequency of 2658 lines/mm were recorded in the photopolymers of Table 1 using the holographic setup of Figure 3a. The obtained results are presented in Figure 4, in which each plot shows the experimental diffraction efficiency (DE) in percentage for the different ACPA concentrations. The reconstruction angle is represented in the horizontal axis, and the origin of the axes corresponds to the Bragg angle for the reconstruction wavelength, that is, an angle of 57.3° for a wavelength of 633 nm as was explained in Section 3.2. Moreover, the theoretical fit of the experimental data with the Coupled Wave Theory [17], explained in Section 3.3., is represented with a solid line in each plot. The experimental spatial period, *Λ_exp_*, the refractive index modulation *Δn*, and the grating thickness *d* [21] are the grating parameters which can be extracted from the fitting. 

The DE obtained with composition 1 without ACPA is represented in Figure 4a. The maximum diffraction efficiency (*DE_Max_*) that was obtained is 42%. A *DE_Max_* of 57% was obtained with composition 2 (ACPA concentration of 0.006 M), and it is represented in Figure 4b. A *DE_Max_* of 72% was obtained with composition 3 (ACPA concentration of 0.009 M), which is represented in Figure 4c. Figure 4d represents the *DE* obtained with composition 4 (ACPA concentration of 0.012 M), for which a *DE_Max_* of 82% was obtained. And finally, Figure 4e depicts the DE obtained with composition 5 (ACPA concentration of 0.0015 M), for which a *DE_Max_* of 81% was obtained. Higher concentrations of ACPA were tested, but the results were not included in this work since *DE_Max_* was not improved.

For a better discussion and comparison of the obtained results, a summary of the main results of Figure 4 is presented in Table 2. One of the main results is that the *DE_Max_* increases as the ACPA concentration in the photopolymer increases, obtaining a *DE_Max_* of 82% with an ACPA concentration of 0.012 M. From this concentration, *DE_Max_* remains constant with the increment of the ACPA concentration. In this sense, the presence of the ACPA in the photopolymer has produced a length reduction in the polymer chains, which has resulted in an increase of the *DE_Max_*.

If our attention is now focused on the refractive index modulation *Δn* and on the optical thickness *d* obtained with the Coupled Wave Theory (Equation (11)), we can see that *Δn* presents small variations between 0.00133 and 0.00154 for the different compositions. On the other hand, the optical thickness *d* increases as the ACPA concentration does, and hence as *DE_Max_* does. This result makes sense since the product *Δn* × *d* is directly related to the diffraction efficiency of a grating [22]. In fact, an increment of the *DE_Max_* produces an increment in the product *Δn* × *d* as can be seen in Table 2. Therefore, if *Δn* remains approximately constant, then *d* must necessarily increase with the increment of the diffraction efficiency of the grating. The observed increment of the optical thickness *d* can be qualitatively explained taking into account that, in the transmission geometry, both recording beams impinge symmetrically at the same sample face. Consequently, both recording beams undergo the same attenuation (due to the dye) at all the spatial positions of the sample where they interfere, and thus the beam ratio of the two beams is close to unity in all of the sample volumes. This fact allows for the ACPA to improve the grating formation in the entire sample volume, which, in turn, explains the increment of the optical thickness associated with the increment of ACPA concentration.

Moreover, the experimental spatial period *Λ_exp_* obtained from the fitting of experimental data of Figure 4 (through Equation (11)) has been represented in Table 2. With these values, the shrinkage can be calculated through Equation (14), taking into account that the theoretical spatial period for these gratings is *Λ_th_* = 0.3762 μm. For all the compositions in Table 2, the values of the shrinkage are smaller than 0.2%, so it can be considered that these gratings are not shrinking. 

**Figure 5:**
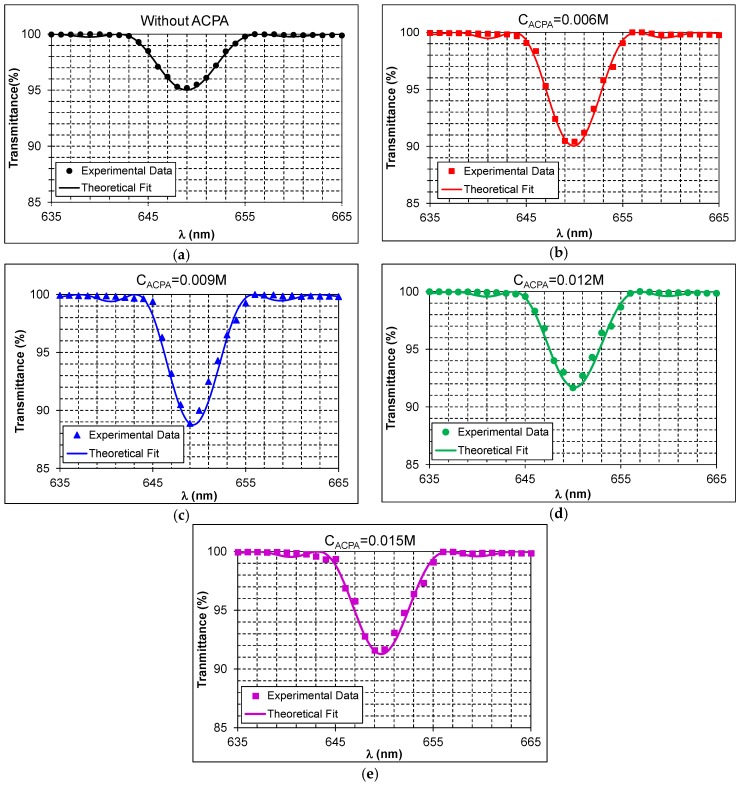
Transmittance as a function of reconstruction wavelength for reflection gratings stored with a spatial frequency of 4558 lines/mm for the compositions of Table 1, (**a**) C1 without ACPA; (**b**) C2 with *C_ACPA_* = 0.006 M; (**c**) C3 with *C_ACPA_* = 0.009 M; (**d**) C4 with *C_ACPA_* = 0.012 M; and (**e**) C5 with *C_ACPA_* = 0.015 M.

Secondly, reflection gratings with a spatial frequency of 4558 lines/mm were recorded in the photopolymers of Table 1 with the setup of Figure 3b, and the obtained results are presented in Figure 5. In this case, each plot shows the experimental transmittance in percentage for a reflection grating with a different ACPA concentration. 

As was explained in Section 3.2, reflection gratings were reconstructed with a double beam spectrophotometer. The reconstruction angle was 0°, and the transmittances of the gratings were obtained as a function of the reconstruction wavelength. Like in Figure 4, the theoretical fit of the experimental data with the Coupled Wave Theory [17] is represented with a solid line in each plot, the spatial period *Λ_exp_*, the refractive index modulation *Δn* as well as the grating thickness *d* are also obtained. In this case, for each plot, *DE_Max_* is calculated from the experimental data by means of Equation (8).

The *DE_Max_* obtained for composition 1 without ACPA was 4.8% (Figure 5a), whereas a *DE_Max_* of 9.6% (Figure 5b) was obtained for composition 2 (ACPA concentration of 0.006 M). On the other hand, a *DE_Max_* of 11.1% (Figure 5c) was obtained for composition 3 (ACPA concentration of 0.009 M), while a *DE_Max_* of 8.3% (Figure 5d) was obtained for composition 4 (ACPA concentration of 0.012 M). Finally, a *DE_Max_* of 8.4% (Figure 5e) was obtained for composition 5 (ACPA concentration of 0.0015 M).

A summary of the results of Figure 5 is presented in Table 3. In this case, the *DE_Max_* increases with the ACPA concentration in the photopolymer until the highest diffraction efficiency (*DE_Max_* of 11.1%) among all compositions of Table 1 is reached for an ACPA concentration of 0.009M. With ACPA concentrations of 0.012 M and 0.015 M, the *DE_Max_* slightly decreases (8.3% and 8.4%, respectively). Once again, the incorporation of the ACPA in the composition of the photopolymer, with the consequent reduction of the length in the polymer chains, produces an increment of the diffraction efficiency.

Regarding *Δn* and d (obtained from the fitting of the experimental data of Figure 5 through Equation (11)), we can see that the optical thickness for reflection gratings remains approximately constant in this case. Consequently, when *DE_Max_* (and therefore the product *Δn* × *d)* increases, *Δn* also does, and when *DE_Max_* and the product *Δn* × *d* decrease, *Δn* also decreases. The fact that the optical thickness remains approximately constant can be qualitatively explained taking into account that, in the reflection geometry, each recording beam impinges symmetrically at a different sample face. Thus, there will be a region inside the sample where both recordings beams undergo approximately the same attenuation and thus the beam ratio of the recording beams will be approximately equal to unity. This fact allows that the ACPA improves the grating formation in that region of the sample. However, outside such region, each of the recording beams will undergo a different attenuation, so the beam ratio will be different from unity, which avoids a suitable interference between the recording beams despite the presence of ACPA. Consequently, the ACPA concentration will only be able to improve the grating formation in a reduced region where the beam ratio is approximately equal to unity, which explains that *d* does not significantly increase as the ACPA concentration does.

Moreover, the experimental spatial period *Λ_exp_* has been obtained from the experimental data of Figure 5. The values calculated from the fitting through Equation (11) have been included in Table 3. Like in the transmission geometry, the shrinkage has also been calculated through Equation (14) taking into account that the theoretical spatial period for these gratings is *Λ_th_* = 0.2194 μm. The values of shrinkage are included in Table 3 (S%). As can be observed, the shrinkage value for the composition without ACPA (*S* = 2.30%) is higher than the shrinkage values for the compositions with ACPA. However, all the shrinkage values range between 2.13% to 2.30%, so we can conclude that the ACPA has not a significant influence on shrinkage.

Finally, from the comparison of Table 2 and Table 3, it can be observed that the values of *Δn* for the reflection gratings are greater than the ones obtained for transmission gratings. However, the *DE* for reflection gratings is smaller than for transmission gratings, since the optical thickness (and thus the product *Δn* × *d)* in the reflection gratings is much smaller than in the transmission ones. In fact, unlike in the transmission geometry, the recording beams in the reflection geometry do not necessarily undergo the same attenuation at the interference region inside the sample. Consequently, the spatial region where the beam ratio is close to unity is much smaller than in the case of transmission gratings. Furthermore, this fact implies that the grating improvement due to the ACPA will be restricted to a much smaller region (where the beam ratio is close to unity) than in the case of transmission gratings, which explains that the optical thickness does not significantly improve with the ACPA.

## 5. Conclusions

In this paper, holographic transmission gratings with a spatial frequency of 2658 lines/mm and holographic reflection gratings with a spatial frequency of 4558 lines/mm were recorded in a PVA/AA based photopolymer. A CTA, specifically ACPA, in this work is incorporated into this material to improve its response at high spatial frequencies. The ACPA concentrations have been modified to determine the optimal composition that allows for obtaining a maximum diffraction efficiency in each geometry. 

For the transmission geometry, it has been obtained that the incorporation of the ACPA into the standard photopolymer produces two times the diffraction efficiency than in the photopolymer without ACPA. Although the presence of ACPA in the material has not significantly improved the refractive index modulation, it has improved the grating formation in the entire volume of the material, which causes an optical thickness increment and, in turn, a diffraction efficiency increment.

For the reflection geometry, the presence of ACPA has also produced twice the diffraction efficiency than in the photopolymer without ACPA. In this case, the optical thickness of the grating has only increased slightly due to the geometry of the recording system. However, the ACPA has improved refractive index modulation, thereby improving the diffraction efficiency of the grating.

Shrinkage has also been calculated for the transmission and reflection gratings. For transmission gratings, the shrinkage was below 0.2%, so it can be considered that such gratings do not have shrinkage. In the case of reflection gratings, the shrinkage was approximately constant, ranging between 2.1% and 2.3% for all the analyzed compositions, and thus the ACPA did not have an appreciable influence on the shrinkage.

## Figures and Tables

**Table 1:** Concentrations (C1, C2, C3, C4 and C5) of the photopolymer compositions.

Component	C1	C2	C3	C4	C5
Polyvinylalcohol (PVA)	8.26% m/v	8.26% m/v	8.26% m/v	8.26% m/v	8.26% m/v
Acrylamide (AA)	0.44 M	0.44 M	0.44 M	0.44 M	0.44 M
Triethanolamine (TEA)	0.20 M	0.20 M	0.20 M	0.20 M	0.20 M
Yellowish eosin (YE)	2.4 × 10^−4^ M	2.4 × 10^−4^ M	2.4 × 10^−4^ M	2.4 × 10^−4^M	2.4 × 10^−4^ M
4,4´-Azobis(4-cyanopentanoic acid) (ACPA)	–	0.006 M	0.009 M	0.012 M	0.015 M

**Table 2:** Parameters obtained from the fitting of the gratings in Figure 4.

*C_ACPA_* (M)	*DE_Max_* (%)	*Δn*	*d* (μm)	*Δn* × *d* (μm)	*Λ_exp_* (μm)
0	42	0.00140	55.9	0.078	0.376818
0.006	57	0.00133	72.5	0.096	0.376836
0.009	72	0.00154	73.5	0.113	0.376853
0.012	82	0.00136	93.7	0.127	0.376486
0.015	81	0.00145	86.9	0.126	0.376329

**Table 3:** Parameters obtained from the fitting of the gratings in Figure 5.

*C_ACPA_* (M)	*DE_Max_* (%)	*Δn*	*d* (μm)	*Δn* × *d* (μm)	*Λ_exp_* (μm)	S (%)
0	4.8	0.00245	19.1	0.047	0.214340	2.30
0.006	9.6	0.00308	21.9	0.068	0.214646	2.16
0.009	11.1	0.00330	21.9	0.072	0.214458	2.25
0.012	8.3	0.00290	21.2	0.061	0.214723	2.13
0.015	8.4	0.00300	21.0	0.063	0.214573	2.19

## Abbreviations

The following abbreviations are used in this manuscript:

PVA: Polyvinyl Alcohol
AA: Acrylamide
ACPA: 4,4’-azobis (4-cyanopentanoic acid)
TEA: Triethanolamine
YE: Yellowish Eosin
CTA: Chain Transfer Agent
*Δn*: Refractive Index Modulation
*d*: Grating Thickness
DE: Diffraction Efficiency
DE_Max_: Maximum Diffraction Efficiency
